# Sensitivity and performance of three novel quantitative assays of SARS-CoV-2 nucleoprotein in blood

**DOI:** 10.1038/s41598-023-29973-3

**Published:** 2023-02-17

**Authors:** Thore Hillig, Josephine R. Kristensen, Claus L. Brasen, Ivan Brandslund, Dorte A. Olsen, Camilla Davidsen, Jonna S. Madsen, Claus A. Jensen, Young B. L. Hansen, Lennart Friis-Hansen

**Affiliations:** 1 Department of Clinical Biochemistry, Nordsjællands Hospital, Dyrehavevej 29, 3400 Hillerød, Denmark; 2 Department of Clinical Biochemistry, Bispebjerg and Frederiksberg Hospitals, Copenhagen, Denmark; 3 Biochemistry and Immunology, University Hospital of Southern Denmark, Odense, Denmark; 4 Department of Regional Health Research, Faculty of Health Sciences, University of Southern Denmark, Odense, Denmark; 5 Department of Clinical Medicine, University of Copenhagen, Copenhagen, Denmark

**Keywords:** Biomarkers, Health care, Medical research, Pathogenesis, Signs and symptoms

## Abstract

To assess if SARS-CoV-2 (COVID-19) systemic disease can be determined by available nucleoprotein assays, we compared the performance of three commercial SARS-CoV-2 nucleoprotein (N) assays in plasma. A total of 272 plasma samples collected in the period November–December 2021 were analyzed by the methods Simoa SARS CoV‐2 N Protein Advantage Kit [Quanterix Simoa], Solsten SARS-CoV-2 Antigen enzyme immunosorbent assay (ELISA) [Solsten ELISA], and Elecsys SARS‐CoV‐2 Antigen electrochemiluminescence immunoassay [Elecsys ECLIA]. Additionally, a dilution series of inactivated virus culture was analyzed by the three assays. The SARS CoV-2 PCR-status was not known for the patients. Linear correlation in the pairwise correlation between assays as well as linearity of dilution series of inactivated virus culture was estimated by Spearman score. Sensitivity and specificity were estimated by pairwise comparison. The three assays showed poor agreement on patient samples with regards to concentration. Performance on virus culture was excellent but with different level of detection (LOD). Positive vs negative results show comparable sensitivity and specificity of Quanterix Simoa and Solsten ELISA, with a higher LOD in Elecsys ECLIA and thus lower sensitivity and high specificity. N by all tested assays can be used as a marker for systemic COVID-19 disease.

## Introduction

The SARS-CoV-2 (COVID-19) nucleoprotein (N) is highly conserved in the COVID-19 genus and the function of the N protein is to package the viral genome RNA into a long helical ribonucleocapsid complex and to participate in the assembly of the virion^1^. The N protein is one of the most abundant structural proteins in virus-infected cells^2^. Therefore, given both its abundance and conservation of the structure many N tests have been developed not only as lateral flow test but also on traditional immunochemical platforms as alternative to traditional NAAT for diagnostic and monitoring purposes^3,4^.

Initially N assays were developed for diagnostic/screening purposes for testing oro/nasopharyngeal swabs from the upper and lower respiratory tracts, and usually with qualitative (ordinal) results. While especially the lateral flow antigen tests are competitive if not superior to the traditional NAAT test with regards to pricing, turnaround time and availability, they are in general less sensitive and have lower specificity but have proven sufficient for mass COVID-19 screening where test sensitivity is secondary to frequency and turnaround time^5^. However, as the state of COVID-19 infection is moving from an epidemic state towards an endemic state and as new therapies are emerging the clinical needs are changing, there is a need for quantitative assessment of viral load by measuring the N concentration in plasma as a tool for guiding therapy e.g. administrating monoclonal antibodies (mAbs) to high risk COVID-19 patients^3,6^. N can be detected during active infection as early as day 2 from symptom onset with a diagnostic sensitivity of 82%^7^. During the first week of the SARS-CoV-2 infection, detection of N in plasma has a sensitivity of > 90% for the infection with concentration positively correlated to disease severity^8^. Quantification of viremia by N in plasma can therefore be used for risk stratification and/or as a prospective marker for poor outcome in COVID-19 patients^4,9–12^. However, quantitative assays should have a sensitivity and specificity needed for answering the clinical question raised e.g. guidance of mAbs therapy. Furthermore, they should have a short turnaround time that support clinical decision making (often less than 6 h). We therefore compared the performance of three commercial N assays. The “Solsten SARS-CoV-2 Antigen ELISA Kit” (Solsten ELISA) from Solsten Diagnostics, a CE-IVD registered quantitative COVID-19 diagnostic assay for analysis of plasma samples. The “Elecsys SARS-CoV-2 Antigen” (Elecsys ECLIA), an electro chemiluminescence immunoassay (ECLIA) from Roche, which is registered for qualitative diagnostic analysis of upper respiratory swab extracts but here used of label as proof of concept for quantitative analysis of plasma samples^13^. It is therefore a laboratory developed test and is used clinically for monitoring patients and determining when they are no more contagious. This is in accordance with the IVD directive for Laboratory developed tests applied on the responsibility of the Laboratory director.

Quanterix Simoa is a quantitative assay for the automated Simoa HD-X platform using Single Molecule Array technology^14^. The assay is recommended for research use only (RUO) on upper respiratory swab extracts but here used off label for quantitative analysis of plasma samples^15^.

Simoa uses the same reagents as conventional ELISA but uses femtoliter-sized reaction chambers approximately 2 billion times smaller than conventional ELISA. This will result in a rapid buildup of fluorescence if a labeled protein is present and making it possible to detect single molecules and offers a sensitivity up to 1000-fold greater than conventional immunoassays. The Simoa N antigen assay was developed for our own use and in collaboration with Quanterix. It is therefore a laboratory developed test and is used clinically for monitoring patients and determining when they are no more contagious. This is in accordance with the IVD directive for Laboratory developed tests applied on the responsibility of the Laboratory director.

## Materials and methods

This retrospective study follows the guideline “standard for reporting of studies of diagnostic accuracy” (STARD criteria)^16^.

Three sites compared the performance of the local N test (Nordsjællands Hospital (Solsten ELISA), Sygehus Lillebælt (Quanterix Simoa) and Bispebjerg Hospital (Elecsys ECLIA)) in the period of November–December 2021. A total of 272 samples were collected and analyzed across the three sites from surplus samples requested by the clinicians for routine biochemical analysis. Samples (lithium-heparin or Ethylenediaminetetraacetic Acid Tetrasodium (EDTA) plasma) were divided in three aliquots (100–300 µL), frozen at − 80 °C and shipped frozen for each site. The collection period was March 2020-November 2021 (before the spread of the COVID-19 Omicron variant). Serum samples were not tested on Quanterix Simoa and Elecsys ECLIA assays. Serum samples can be used on the Solsten ELISA but were not in the current study.

Additionally, a dilution series (0.055-1000 Plaque Forming Unit (PFU)/mL) of inactivated virus culture was distributed and measured by the three assays.

The measurements were performed according to manufacturer’s respective instructions with the following adjustments:

Solsten ELISA (Solsten Diagnostics, Aarhus, Denmark) was applied to a BEP2000 Advance automated ELISA platform (Siemens AG, Munich Germany) in which 50 µL of the samples were measured as previously described^13^. The measurement range was 2–250 ng/L, with results > 250 ng/L rerun on diluted samples. The cutoff (manufacturer’s instructions) for a positive result was 10 ng/L.

Quanterix Simoa (Quanterix, Billerica, MA, USA) was applied on the automated Simoa HD-X Analyzer platform (Quanterix). The measurement range was 0.10–100,000 ng/L. Results above 1000 ng/L were diluted up to 100× and rerun. The cutoff for a positive result was 0.15 ng/L.

**Figure 1:**
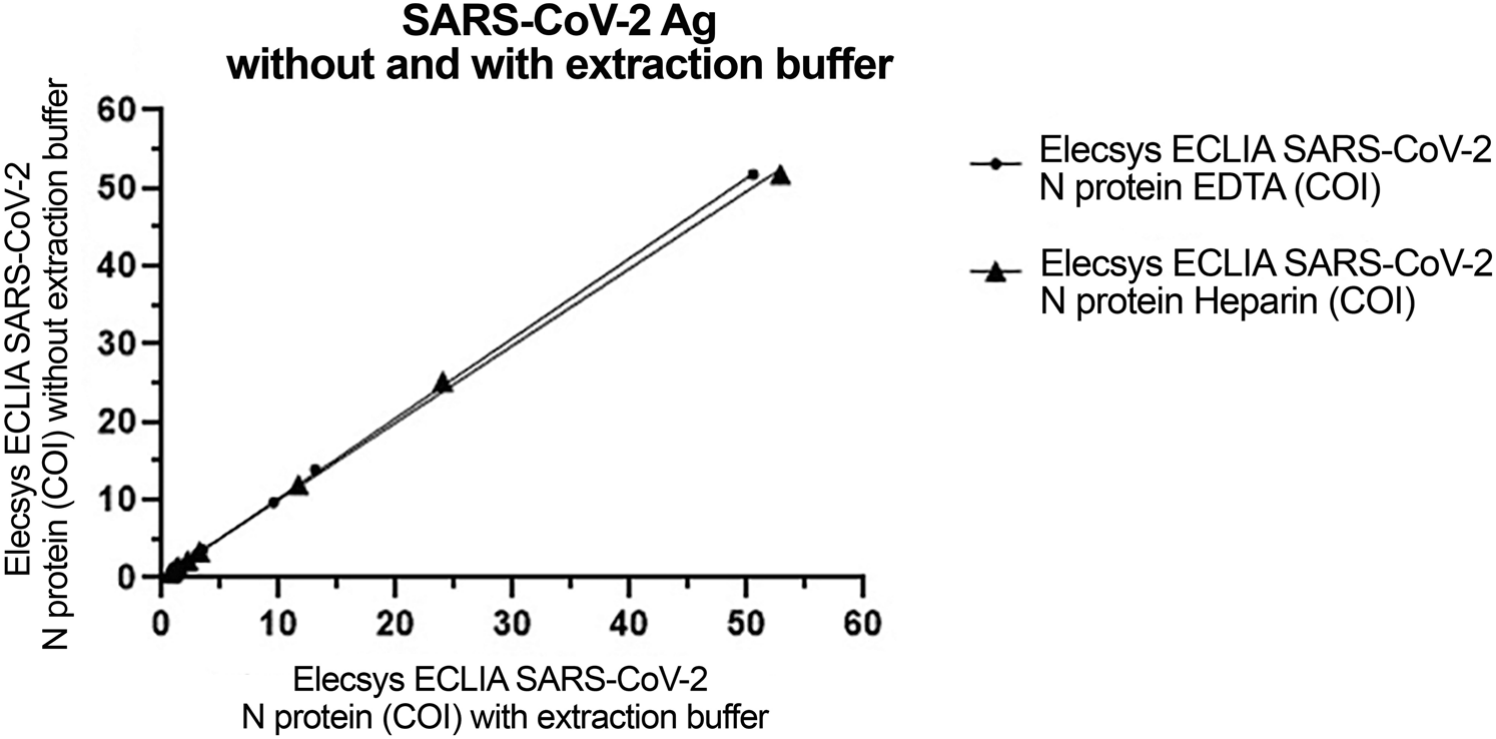
Elecsys ECLIA assay linearity with and without extraction buffer in 10 positive and 10 negative matched EDTA and Heparin plasma samples.

Elecsys ECLIA (Roche Diagnostics Gmbh, Mannheim, Germany) was performed on a Cobas^®^ e801 analyzer (Roche Diagnostics Gmbh, Mannheim, Germany). The measurement range was 0.25-160 CutOff Index (COI, a measurement unit defined by Roche). The factory set cutoff is 1.00 for oro/nasopharyngeal swab and the assay is to be used for nasopharyngeal/oropharyngeal swab that are either transferred to an 1× extraction buffer or UTM media that is extracted with an 10× extraction buffer and not designed for testing for N in plasma. To determine the background in plasma 36 matched EDTA and heparin samples were tested, and EDTA and heparin samples were tested with and without extraction adding 1/10 10× extraction buffer. Limit of blank (LOB) was calculated LOB = mean + 1.645*SD and Limit of Detection (LOD) was calculated LOD = mean + 3*SD^17^. The performance of the assay with and without extraction buffer was tested on 10 N positive Heparin samples and 10 N positive EDTA samples (Fig. 1).

Data was log-transformed but not normal distributed and analyzed by Deming correlations (Spearman, r-value) compared across all assays, pairwise. Contingency tables for all assays pairwise was analyzed for sensitivity and specificity as well as comparison by Fisher’s Exact test, Cohens Kappa and McNemar test.

Statistical analyses were performed using GraphPad Prism 9.4 (GraphPad Systems). P values < 0.05 were considered statistically significant.

### Power analysis

The power and intended sample size were estimated a priori as requested by the STARD. With estimated sensitivity and specificity of 80%, a prevalence of 50%, the intended minimum subjects with an error < 5% was estimated to 28 subjects. The study was carried out with collection of more than the required number of samples.

### Ethics

For the purpose of quality control and method comparisons left over surplus serum and plasma material obtained from the daily clinic at the Clinical Biochemistry Departments of Nordsjælland Hospital, Sygehus Lillebælt and Bispebjerg Hospital were collected and stored in a clinical biobank after anonymization. The study was performed according to the Danish data protection laws and EU GDPR. According to the Danish health legislation § 42 no informed consent from patients are needed in such kind of quality and method comparisons performed on anonymized surplus diagnostic material with no clinical or personal data^18^. The present quality control study of the N test method did not imply extra blood sampling from the patients. The obtained results had no impact on clinical care decisions, and no clinical information was collected. The study complied with all relevant national regulations, institutional policies and is in accordance with the tenets of the Helsinki Declaration (as revised in 2013). Accordingly, the present quality control study of three methods required no written or oral permission from the patients^18^.

## Results

### Adaption of the Elecsys SARS-CoV-2 Elecsys ECLIA assay for plasma measurements

LOB was 0.33 and the LOD was 0.35–0.38, therefore a cutoff > 0.40 COI for a positive result was chosen. There was no difference in LOB and LOD between heparin or EDTA samples with and without addition of extraction (Table 1). Twenty samples from infected patients were subsequently tested demonstrating values above the cutoff and identical values with and without extraction buffer for both EDTA and heparin samples (Table 2). It was concluded that the assay could measure N in EDTA/heparin plasma without extraction and that the assay should be calibrated against known quantitative assays.

**Table 1:** Determination of the limit of detection of the N-antigen for the Elecsys ECLIA assay.

Sample type	EDTA + extraction buffer	EDTA without	Heparin + extraction buffer	Heparin without
Number of samples	36	36	36	36
Median, (range)	0.29 (0.26 to 0.40)	0.27 (0.21 to 0.38)	0.32 (0.31 to 0.38)	0.31 (0.30 to 0.34)
Std. deviation	0.029	0.028	0.012	0.010
Limit of blank	0.34	0.34	0.33	0.33
Limit of detection	0.38	0.36	0.37	0.35
Cutoff (decided)	< 0.40	< 0.40	< 0.40	< 0.40

**Table 2:** Comparison of Elecsys ECLIA N-antigen measurements in EDTA/Heparin plasma without vs with addition of the extraction buffer. (95% confidence intervals).

Sample type	EDTA without vs with extraction buffer	Heparin without vs with extraction buffer
Number of samples	10	10
Slope	1.03 (0.96 to 1.1)	0.99 (0.86 to 1.1)
Y-intercept	− 0.046 (− 0.31 to 0.22)	0.14 (− 0.41 to 0.68)
Spearman r	> 0.99	> 0.99

**Figure 2:**
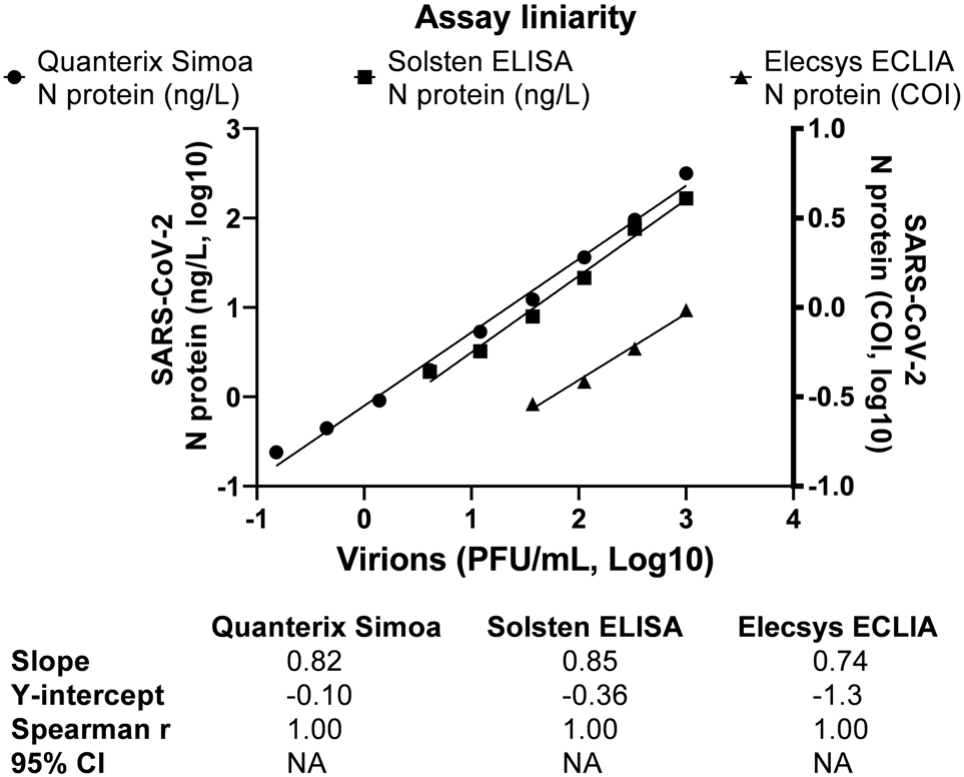
Assay linearity in a dilution series (0.055–1000 PFU/mL) of inactivated virus. The Quanterix Simoa and Solsten ELISA assays use the unit ng/L (left y-axis). The Elecsys ECLIA assay use the unit COI (right Y-axis).

In the virus culture dilution series in the range of 0.055–1000 PFU/mL, the Quanterix Simoa, Solsten ELISA and Elecsys ECLIA assays showed calibration slopes (double log-scale) of 0.821, 0.854 and 0.743, respectively (Fig. 2). Linearity for full range of the virus culture was excellent (Spearman > 0.99) for all assays (Fig. 2). The lowest titer of the virus culture detected was 0.15 PFU/mL for Quanterix Simoa (0.24 ng/L), 4.1 PFU/mL for Solsten ELISA (2.0 ng/L) and 37 PFU/mL for Elecsys ECLIA assays (0.84 COI which equals 12 ng/L when measured using the Quanterix Simoa assay).

**Figure 3:**
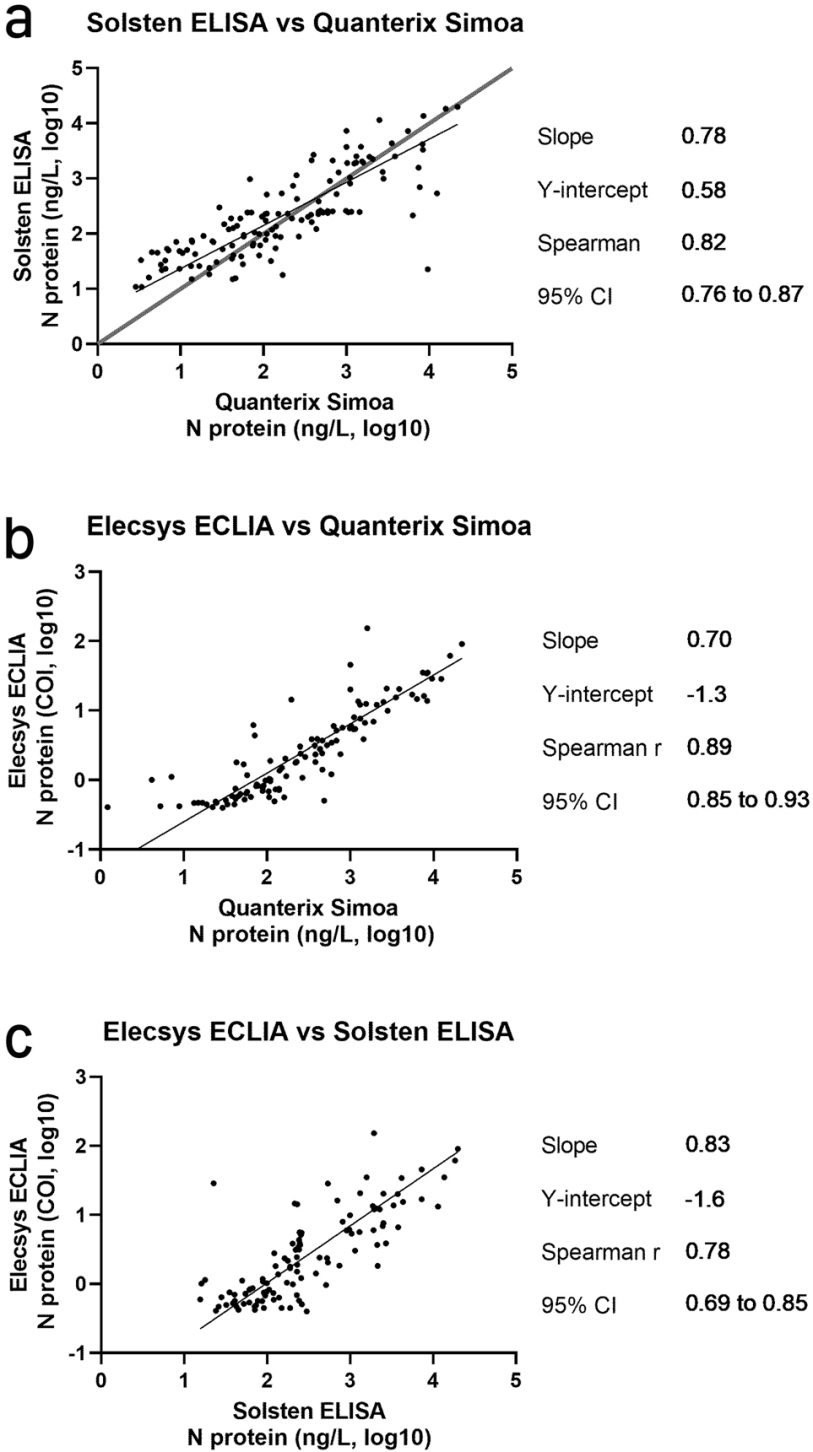
Pairwise assay comparisons of Solsten ELISA and Quanterix Simoa (**a**), Elecsys ECLIA and Quanterix Simoa (**b**) and Elecsys ECLIA and Solsten ELISA (**c**).

Linear correlation in the pairwise correlation between assays (Fig. 3) was estimated by Spearman score (Quanterix Simoa vs Solsten ELISA, Spearman ≈ 0.82), (Quanterix Simoa vs Elecsys ECLIA, Spearman ≈0.89), (Solsten ELISA vs Elecsys ECLIA, Spearman ≈ 0.78).

Contingency tables for Quanterix Simoa vs Elecsys ECLIA and Elecsys ECLIA vs Quanterix Simoa showed sensitivities of 75.5% and 100.0%, respectively and specificities of 100.0% and 77.6%, respectively (Table 3a). Contingency tables between Solsten ELISA vs Elecsys ECLIA and Elecsys ECLIA vs Solsten ELISA showed sensitivities of 74.8% and 99.1%, respectively and specificities of 99.2% and 77.0%, respectively (Table 3b). Contingency tables of Quanterix Simoa vs Solsten ELISA and Solsten ELISA vs Quanterix Simoa showed similar sensitivities of 87.8% and similar specificities of 85.6% (Table 3c). The contingency tables between Quanterix Simoa vs Elecsys ECLIA, Solsten ELISA vs Elecsys ECLIA and Quanterix Simoa vs Solsten ELISA,and were analysed for Fishers exact test (P < 0.0001, P < 0.0001 and P < 0.0001, respectively), Cohens Kappa (0.74, 0.72 and 0.73, respectively) and McNemars test ( P < 0.0001, P < 0.0001 and P = 0.87, respectively).

**Table 3:** 2 × 2 contingency tables between each assays with resulting diagnostic performance with each assay set as truth.

a	Quanterix Simoa				Solsten ELISA		
Solsten ELISA		pos	neg	Quanterix Simoa		pos	neg
	pos	129	18		pos	129	18
	neg	18	107		neg	18	107
Sensitivity	87.8%		81.3% to 92.6%	Sensitivity	87.8%		81.3% to 92.6%
Specificity	85.6%		78.2% to 91.2%	Specificity	85.6%		78.2% to 91.2%

b	Quanterix Simoa				Elecsys ECLIA		
Elecsys ECLIA		pos	neg	Quanterix Simoa		pos	neg
	pos	111	0		pos	111	36
	neg	36	125		neg	0	125
Sensitivity	75.5%		67.7% to 82.2%	Sensitivity	100.0%		96.7 to 100.0%
Specificity	100.0%		97.1% to 100.0%	Specificity	77.6%		70.4 to 83.8%

c	Solsten ELISA				Elecsys ECLIA		
Elecsys ECLIA		pos	neg	Solsten ELISA		pos	neg
	pos	110	1		pos	110	37
	neg	37	124		neg	1	124
Sensitivity	74.8%		67.0% to 81.6%	Sensitivity	99.1%		95.1% to 100.0%
Specificity	99.2%		95.6% to 100.0%	Specificity	77.0%		69.7% to 83.3%

Comparison	Fisher's exact test*	Cohens Kappa**	McNemar***				
Quanterix Simoa vs Solsten ELISA	< 0.00001	0.73	0.87				
Quanterix Simoa vs Elecsys ECLIA	< 0.00001	0.74	< 0.0001				
Solsten ELISA vs Elecsys ECLIA	< 0.00001	0.72	< 0.0001				

*P-value.

**Kappa value, substantial agreement.

***P-value.

## Discussion

We have compared N measurement performance from three different commercial assays: Quanterix Simoa, Elecsys ECLIA and Solsten ELISA. We found all three assays had an excellent performance in a virus culture dilution series (Spearman r > 0.99), however with different LOD, lowest by Quanterix Simoa, highest by Elecsys ECLIA. In patient samples, the measured concentrations of N were different in the three assays (Spearman 0.78–0.89). This non-linearity is often seen when using patient samples in immunoassays due to interfering matrix effects from plasma. There is yet no established NIST calibrator and thus concentrations and cutoffs are difficult to compare for each assay. However, the discriminatory power of each assay to find positive and negative patients are more comparable. Without a true result, e.g. a PCR confirmed diagnosis, it is not possible to discern which result is truer, when two assays differ in the category “N positive” or “N negative”. Thus, we have made pairwise comparisons of all 272 results from Quanterix Simoa vs Solsten ELISA, Quanterix Simoa vs Elecsys ECLIA, Solsten ELISA vs Elecsys ECLIA. The resulting six 2 × 2 contingency tables are depicted in Table 3.

Comparing Quanterix Simoa and Elecsys ECLIA (Table 3a), all discrepant results (36) are negative in Elecsys ECLIA and positive in Quanterix Simoa, pointing to a reduced sensitivity of the Elecsys ECLIA assay. The Fisher’s exact test points to difference between assays (P < 0.0001), as does the McNemar test (P < 0.0001), however Cohens Kappa show substantial agreement (0.74).

Comparing Solsten ELISA and Elecsys ECLIA (Table 3b), most discrepant results (37 of 38) are negative in Elecsys ECLIA and positive in Solsten, pointing to a reduced sensitivity of the Elecsys ECLIA assay. The Fisher’s exact test points to difference between assays (P < 0.0001), as does the McNemar test (P < 0.0001), however Cohens Kappa show substantial agreement (0.72).

Comparing Quanterix Simoa and Solsten ELISA (Table 3c), there are an equal number (18) of samples in the discrepant categories. Either this points to the two assays finding equal numbers but not exactly the same SARS-CoV-2 positive patients or one assay is superior with regards to both sensitivity and specificity. However, since the comparison with the Elecsys ECLIA assay is similar for the two assays the prior explanation seems most plausible. This conclusion is supported by the accompanying statistical analysis, where although the Fisher’s exact test points to difference between assays (P < 0.0001), both the McNemar test (P = 0.87) and Cohens Kappa show substantial agreement (0.73).

Of the three assays, the Quanterix Simoa stands out with a lower limit of detection and larger measuring range. This gives rise to some disparity in results for the lowest and highest concentrations of N.

The Elecsys ECLIA assay stands out with the highest LOD, which results in samples with concentration of N below LOD being detected as negative and thus a lower sensitivity.

However, the current study does not have a verified truth to compare to, thus the base conclusion of the comparison is that each assay is different. The performance with regards to sensitivity/specificity, positive and negative predictive values should be evaluated for each assay, preferably vs gold standard methodology (NAAT-test) for each defined purpose (screening, de-isolation, neutralizing monoclonal antibody treatment) as was done previously for the Solsten ELISA^13^ and Quanterix Simoa^3^.

The Elecsys ECLIA assay was tested in an off-label use of plasma/serum vs the intended use of saliva or nasal mucus. However, we performed internal control of the assay (Tables 1, 2) and found excellent correlation (Table 2, Spearman > 0.99) between EDTA/heparin-plasma with or without addition of extraction buffer. Thus, we showed that plasma samples can be measured by the Elecsys ECLIA assay.

The primary reason for testing the Elecsys ECLIA assay is the routine high-throughput applicability, in contrast to the Quanterix Simoa and Solsten ELISA assay, which currently have issues in relation to communication with Laboratory Information systems (LIMS) and thus depend on manual procedures of reporting.

Also, the Elecsys ECLIA assay is a random-access test with a turnaround time of less than 60 min. In contrast, the Quanterix Simoa and Solsten ELISA assays are analyzed in batches with a turnaround time of 180 min. depending on demand/test numbers they are therefore often only performed once daily.

Even though cutoff of the Elecsys ECLIA assay was around 0.40 COI (12 ng/L, approximated to Quanterix Simoa results from testing the viral culture) we believe it can still be effectively used in the clinical risk stratification of patients with Covid-19 treated with mAbs as a study using the Quanterix Simoa assay found that those with N > 1000 ng/L benefitted the most^6^. This correlate to approximately 7 COI (1000 ng/L) in the Elecsys ECLIA assay, well above the estimated cutoff of 0.40 COI (12 ng/L). In the Solsten ELISA assay, a Quanterix Simoa result of 1000 ng/L corresponds to approximately 855 ng/L, again well above the cutoff of 10.0 ng/L. Thus, each of the tested assays should be suitable for use in selection of patients eligible for neutralizing antibody treatment.

Presence of N in plasma is, at least in part, a marker for active or recently active viral replication in the patient and maybe a better marker for viral replication than NAAT since N is associated with cellular membranes and are nuclease resistant^4^. They can therefore be detected even after active replication has stopped^19,20^. Detection of N in blood can also be used for rapid screening of patients for COVID-19 e.g. in the emergency department, since N is only produced by patients suffering from a limited range of diseases^1,12^. Antigenimia as measured by N can be used as a clinical tool to find the most critical patients and the patients eligible for mAbs treatment^8–11^. Also, N has been observed to appear in urine of COVID-19 patients as a strong marker for serious systemic disease^21^. Currently both the Quanterix Simoa and Solsten ELISA assays have been tested on larger cohorts of patients, retrospectively^3,4,13^.

The main limitations of this study are the lack of gold standard NAAT verification, the lack of international NIST calibrator and off label use of assays for Quanterix Simoa and Elecsys ECLIA.

In summary, three N assays (Quanterix Simoa, Solsten ELISA and Elecsys ECLIA), were compared with all assays showing excellent, albeit different, linearity in a standardized inactivated SARS-CoV-2 virus preparation.

For specific use in the indication treatment with mAbs, the Quanterix Simoa N assay has been reported with a positive effect in patients in concentrations > 1000 ng/L^6^. Corresponding concentrations for the Solsten ELISA and Elecsys ECLIA assays are 855 ng/L and 7 COI (1000 ng/L), respectively. However, each assay should be independently evaluated for specific indications, e.g. screening, monitoring, eligibility for neutralizing antibody treatment, etc. The dataset used in the study is available as (Supplementary Information).

## Supplementary Information

Supplementary Information.

## Data Availability

The datasets used and/or analysed during the current study available from the corresponding author on reasonable request.

## Abbreviations

N: SARS-CoV-2 nucleoprotein
COVID-19: SARS-CoV-2
NAAT: Nucleic Acid Amplification Test
mAbs: Monoclonal antibodies
Solsten ELISA: Solsten SARS-CoV-2 Antigen Enzyme Linked ImmunoSorbent Assay Kit
Elecsys ECLIA: Elecsys SARS‐CoV‐2 Antigen ElectroChemiLuminescence ImmunoAssay
Quanterix Simoa: Simoa SARS CoV‐2 N Protein Advantage Kit
STARD: Standard for reporting of studies of diagnostic accuracy
COI, a measurement unit defined by Roche: CutOff Index
EDTA: Ethylenediaminetetraacetic acid tetrasodium
PFU: Plaque forming unit
LOB: Limit of blank
LOD: Level of detection
